# Improving social function with real-world social-cognitive remediation in schizophrenia: Results from the RemedRugby quasi-experimental trial

**DOI:** 10.1192/j.eurpsy.2020.42

**Published:** 2020-04-30

**Authors:** Julien Dubreucq, Franck Gabayet, Bernard Ycart, Megane Faraldo, Fanny Melis, Thierry Lucas, Benjamin Arnaud, Mickael Bacconnier, Motassem Bakri, Gentiane Cambier, Florian Carmona, Isabelle Chereau, Titaua Challe, Sophie Morel, Sylvie Pires, Celine Roussel, Philippe Lamy, Guillaume Legrand, Emmanuelle Pages, Romain Pommier, Romain Rey, Yohan Souchet, Pierre-Michel Llorca, Catherine Massoubre

**Affiliations:** 1 Centre de Neurosciences Cognitive, UMR 5229, CNRS and Université Lyon 1, Lyon, France; 2 Centre Référent de Réhabilitation Psychosociale et de Remédiation cognitive (C3R), Centre Hospitalier Alpes Isère, Grenoble, France; 3 Fondation FondaMental, Créteil, France; 4 Réseau Handicap Psychique, Grenoble, France; 5 Laboratoire Jean Kuntzmann, CNRS UMR 5224, Université Grenoble-Alpes, Grenoble, France; 6 Centre Hospitalier Sainte Marie de Clermont Ferrand, 33 rue Gabriel Péri, CS 9912, Clermont-Ferrand Cedex 1 63037, France; 7 Centre Médical La Teppe, 25 Avenue de la Bouterne, CS 9721, Tain-l'Hermitage Cedex 26602, France; 8 Centre de Réhabilitation Psychosociale et de Remédiation Cognitive (C2R), CH Drôme Vivarais, Montéléger, France; 9 Centre Hospitalier de la Savoie, 89 avenue de Bassens, Bassens 73000, France; 10 REHALise, CHU de Saint-Etienne, Saint-Etienne, France; 11 CMP B, CHU, EA 7280 Faculté de Médecine, Université d'Auvergne, BP 69, Clermont-Ferrand Cedex 1 63003, France; 12 Centre Départemental de Réhabilitation Psychosociale des Glières, 219 Chemin des Bois des Fornets, La Roche sur Foron 74800, France; 13 Université Claude Bernard Lyon 1/Centre Hospitalier Le Vinatier Pole Est BP 300 39, 95 bd Pinel, Bron Cedex 69678, France

**Keywords:** Physical activity, schizophrenia, social cognitive remediation, social function, touchrugby

## Abstract

**Background.:**

Functional capacity (FC) has been identified as a key outcome to improve real-world functioning in schizophrenia. FC is influenced by cognitive impairments, negative symptoms, self-stigma and reduced physical activity (PA). Psychosocial interventions targeting FC are still under-developed.

**Methods.:**

we conducted a quasi-experimental study evaluating the effects of an exercise-enriched integrated social cognitive remediation (SCR) intervention (RemedRugby [RR]) compared with an active control group practicing Touch Rugby (TR). To our knowledge, this is the first trial to date evaluating the effectiveness of such a program provided in a real-life environment.

**Results.:**

Eighty-seven people with schizophrenia were included and allocated to either the RR group (*n* = 57) or the TR group (*n* = 30) according to the routine clinical practice of the recruiting center. Outcomes were evaluated at baseline and post-treatment in both groups and after 6 months of follow-up in the RR group using standardized scales for symptom severity, social functioning, self-stigma, and a large cognitive battery. After treatment we observed moderate to large improvements in social function (Personal and Social Performance Scale [PSP], *p* < 0.001, *d* = 1.255), symptom severity (Positive and Negative Syndrome Scale [PANSS] negative, *p* < 0.001, *d* = 0.827; PANSS GP, *p* < 0.001, *d* = 0.991; PANSS positive, *p* = 0.009, *d* = 0.594), verbal abstraction (*p* = 0.008, *d* = 0.554), aggression bias (*p* = 0.008, *d* = 0.627), and self-stigma (stereotype endorsement, *p* = 0.019, *d* = 0.495; discrimination experiences, *p* = 0.047; *d* = 0.389) that were specific to the RR group and were not observed in participants playing only TR. Effects were persistent over time and even larger between post-treatment and follow-up.

**Conclusions.:**

Exercise-enriched integrated SCR appears promising to improve real-life functioning in schizophrenia. Future research should investigate the potential effects of this intervention on neuroplasticity and physical fitness.

## Introduction

Schizophrenia is a severe mental illness ranking among the leading causes of disability across the world. It is associated with decreased functional capacity, impaired real-life functioning [1,2], reduced life expectancy [3] and important societal cost [4]. The combination of optimal pharmacological treatment with psychiatric rehabilitation is recommended in major international guidelines to improve the prognosis of schizophrenia [5,6]. Psychiatric rehabilitation (PR) brings together a wide range of recovery-oriented interventions [7]. They include psycho education, social skills training (SST), cognitive remediation (CR), cognitive behavior therapy (CBT), metacognitive training, improvements in physical and mental health, and supported housing or employment [7,8]. The aim of PR is to promote clinical and personal recovery in patients with schizophrenia. Clinical recovery refers to symptom remission and improved psychosocial functioning during at least 2 years of follow-up [9]. Personal recovery refers to a self-broadening process aiming at living a meaningful life beyond mental illness [10]. Despite improvements in pharmacological treatments and psychosocial interventions, the proportion of patients with schizophrenia meeting the criteria for clinical recovery remains limited (13.5%) [9]. One potential explanation is the complexity of real-world functioning in schizophrenia [1,2]. Real-world functioning results from the interplay of multiple variables, including impairments in cognition and social cognition, negative symptoms, self-stigma, poor physical health, reduced physical activity, and low social support [1–3,11]. It has been suggested to target specifically functional capacity—or one’s abilities to perform in the domains of residential functioning, work, leisure activities and social skills [1]—in PR to improve its effectiveness on real-life functioning [2].

Social cognitive impairments are common and closely related to real-world functioning [11]. Social cognitive remediation (SCR) improves theory of mind (*d* = 0.70), emotion recognition (*d* = 0.84), social perception (*d* = 1.29), and attributional bias (*d* = 0.30–0.52) [12]. SST improves social skills (*d* = 0.52) [13], community functioning (*d* = 0.52) and negative symptoms (*d* = 0.40). Combining SST and SCR would further improve functional outcomes [14]. SCR and SST are however still provided in artificial learning environments using non-ecological materials (comic strips, video scenes for SCR [12], and role-playing for SST [13]). The generalization of treatment benefits to real-life interactions remains therefore limited and enriched environment interventions are needed [11]. Physical activity (PA) is an enriched environment enhancing the CR’s effects on patient’s outcomes [15,16]. CR combined with aerobic exercises has resulted in larger improvements in cognitive function, negative symptoms and social functioning in two pilot studies [15,16]. Individual physical activities (i.e., yoga and aerobic exercises) [17], increasingly used as add-on therapies for people with schizophrenia, have improved physical fitness, positive and negative symptoms, depression, working memory, social cognition, attention, and everyday functioning [17,18]. Low self-efficacy, negative symptoms, depression, social anxiety, social isolation, and lack of social support are potential barriers to PA participation [19–22]. Soccer has been used as an add-on PA with preliminary effects on physical fitness and quality of life [23]. SCR and SST combined with a collective physical activity could improve real-life functioning through improved social cognition and social skills, negative symptoms, social anxiety, and isolation [11,12]. To the best of our knowledge, such an intervention has however not been yet been developed.

The present intervention, RemedRugby (RR), is a structured, manualized 15-session program integrating psychoeducation about stigma and self-stigma, SCR, SST (on the field and with the medias), cognitive remediation targeting executive functions, problem-solving skills training and practice of Touch Rugby (TR). It is based on two assumptions: (a) an intervention provided directly in a real-life environment will improve treatment effectiveness; (b) improvements in social cognition, social skills, and self-stigma will increase engagement in the physical activity, social motivation, and social functioning. Reducing the impact of potential psychological or socio-ecological barriers to PA (i.e., negative symptoms, social anxiety, depression, low self-efficacy, social isolation, and lack of social support [19–22]) could make it more enjoyable and enhance the motivation to participate. Rugby is a popular sport in Western Europe, South Africa and Oceania, with core values of respect, teamwork, enjoyment, discipline, and sportsmanship [24]. Touch Rugby is a limited-contact version of rugby in which players seek to evade being touched (rather than tackled) while in possession of the ball. It is played by mixed teams of five players with easy-to-learn rules that can be mastered even by participants with cognitive impairments and no prior experience of rugby. Sessions can be adapted to participant’s physical health and fitness. Choosing this sport as the support of intervention aims at associating the benefits associated to PA with an increased motivation to engage in social behavior during the sessions and at home. The aims of this quasi-experimental study are to assess RR effectiveness (in comparison with an active control group practicing only TR): (a) on social function; (b) on social cognition, symptoms severity, executive functioning, self-esteem, self-stigma, empowerment, and personal recovery; and (c) whether effects on social function, social cognition, and other outcomes are persisting after 6 months of follow-up.

## Materials and Methods

### Trial design and participants

This study was a controlled, quasi-experimental, multi-centric, prospective, interventional, and exploratory trial conducted between November 2014 and December 2017 in six psychiatric rehabilitation centers (Grenoble, La Roche sur Foron, Valence, Lyon, Clermont-Ferrand, and Saint-Etienne), 1-day hospital (Chambéry) and three medico-social supported living services (Cotagon center, Chardon Bleu and ALPHI homes) located in the Auvergne-Rhône-Alpes region in France. Eligible participants were all 18–65 years-old patients meeting the DSM V criteria for schizophrenia spectrum disorders enough physically fit to practice a PA (determined by a physical examination by patient’s general practitioner) and willing to give informed consent. Exclusion criteria were: (a) a known neurological disorder; (b) intellectual disability; (c) inability to read or speak French; and (d) inability to practice a PA. Participation to other programs having an impact on social function (SCR and SST) was prohibited during the follow-up period. Eighty-seven clinically stable (i.e., absence of significant changes in pharmacological treatment in the past month) participants diagnosed with schizophrenia were consecutively recruited and assessed at baseline, post-treatment (4 months) and at 6 months of follow-up (for RR patients only). The control group was not assessed at follow-up to allow the participation to other interventions improving social function. Forty-seven males and 10 females participated to the RR group, 23 males, and 7 females to the TR group (homogeneity male–female in the access to the intervention, *p* = 0.575 using Fisher’s exact test). Participant’s mean age was 33.3 in the RR group, 33.1 in the TR group. Baseline characteristics of the participants allocated to the two arms are presented in Table 1. The withdrawal of one control center (Bourg en Bresse, reason invoked = lack of time) before the beginning of the study explains the difference in the sample sizes. Psychoeducation about the illness was provided to three RR participants and supportive group psychotherapy to one TR participant during the treatment period or follow-up, with no incidence on treatment’s effectiveness. Participants were allocated in the experimental or the control group depending on the routine clinical practice of the recruiting center. Centers practicing social cognitive remediation animated the experimental group (Grenoble, Lyon, Valence, La Roche sur Foron, and Clermont-Ferrand) and centers practicing PA under the supervision of a specialized sport educator animated the control group (Cotagon, Chardon Bleu, ALPHI, Chambéry, and Saint-Etienne). Experimental centers did not have specialized sport educators and control centers could not implement the experimental intervention (absence or lack of CR-trained facilitators). This made impossible single-blind randomization. Evaluators who did not take part into treatment and blind to the time of assessment conducted baseline and follow-up assessments. All the clinicians involved in the experimental group attended to 1-day training event in RemedRugby, led by authors J.D., F.G., M.F., and F.M. Training included discussion and role-playing exercises, with corrective feedback from the trainers. The manual was comprehensive giving the detail of each session. Regular group supervisions were organized to ensure treatment fidelity. Changes in pharmacological treatment and all the interventions received during the follow-up period were systematically collected for both groups. The trial protocol was in accordance with the Declaration of Helsinki. It was approved by the local ethics committee (CPP Sud-Est V) under the number 2014-002655-26 and registered at www.clinicaltrials.gov (NCT03775564). Written informed consent was sought for all participants.

**Table 1.** Baseline characteristics in the RR and the TR groups

	RR (*n* = 57)		TR (*n* = 30)		Welsh two-sample *t*-test		
Variable	*m*1	*sd*1	*m*0	*sd*0	*T*	*df*	PV
Age	33.3	8.8	33.1	7.9	0.1	65.3	0.901
Education	11.3	2.3	10.7	2	1.3	65.2	0.199
Participation	0.9	0.1	0.9	0.1	−1.7	55.4	**0.093**
PANSS.Tot	76.1	15.3	68.2	14.7	2.3	58.3	**0.022**
PANSS.Pos	15.7	4.7	14.8	4.5	0.8	58.5	0.428
PANSS.Neg	23.4	6.1	19.4	5.7	3	59.8	**0.004**
PANSS.GP	37.3	8.1	33.9	8.7	1.7	52.9	**0.089**
PSP	55.9	12.4	54.9	16.2	0.3	45.2	0.765
ERF-CS.Tot	48.2	25.8	48.2	34.2	0	41.2	0.996
Simil.NB	20.2	5.6	19.8	5.9	0.3	56.9	0.793
BEMRI.NB	5.9	2.5	5.8	2.8	0.2	52.9	0.862
BEMRD.NB	5.4	2.7	5.6	3	−0.3	54.2	0.746
TMTA.Time	41.8	16.4	45.5	24	−0.7	44	0.459
TMTB.Time	114.9	67.8	136.7	94.4	−1.1	43.4	0.274
Coding.NB	50.7	13.1	51.8	14.8	−0.3	53.5	0.732
PerSo.Tot	21.4	4.4	19.7	4.3	1.7	61.3	0.09
Shopping.time	5.8	3.3	7.1	5.3	−1.2	41.6	0.226
MASC.CM	22	7.3	22.7	8.4	−0.4	51	0.717
MASC.Hy	6.2	3.5	7.6	2.9	−2	67.9	**0.048**
MASC.Ho	11.6	5.3	10.1	4.8	1.2	62.1	0.224
MASC.Abs	5.3	3.5	4.6	3	1	65.2	0.338
f.NART	99.8	8.5	100.2	9.2	−0.2	55.5	0.861
ACSo.Tot	31.8	9.5	31.5	8.2	0.2	67.3	0.872
SERS.posit	41.6	10.4	40.9	12.2	0.3	52.3	0.788
SERS.negat	34.1	10.1	37.4	12.9	−1.2	49.1	0.243
QCAE.tot	83.6	9.8	84.5	8.9	−0.4	64.4	0.657
ISMI.Tot	2.3	0.4	2.3	0.6	−0.4	42.3	0.69
ISMI.Alien	2.5	0.7	2.6	0.9	−0.7	47.6	0.471
ISMI.Stereo	2.1	0.5	2	0.7	0.3	46.7	0.75
ISMI.Discri	2.3	0.6	2.4	0.9	−0.5	43	0.635
ISMI.Rsoc	2.4	0.6	2.5	0.8	−0.5	48.9	0.605
ISMI.Rstig	2.3	0.5	2.3	0.5	0.2	58.5	0.847
AIHQ.Host	1.8	0.8	1.9	0.6	−0.9	69.3	0.351
AIHQ.Intent	3	1.1	3.2	1.1	−0.6	59.1	0.529
AIHQ.Anger	2.8	1.2	3.2	1.1	−1.2	58.3	0.238
AIHQ.Blame	3.2	1.2	3.3	1.1	−0.4	62.9	0.7
AIHQ.Agres	1.5	0.5	1.6	0.4	−0.6	72.3	0.519
AIHQ.AttRe	3	1	3.2	1	−0.8	58.2	0.42
STORI.Mora	19.4	8.4	19.5	9.8	0	50.1	0.963
STORI.Cons	26.9	9.9	27.2	11.9	−0.1	48.6	0.907
STORI.Prep	29.2	9.2	29.9	8.8	−0.3	59.6	0.732
STORI.Reco	31	8.5	34.8	9.2	−1.9	53.3	0.069
STORI.Croi	31	9	34.3	10	−1.5	52	0.14
BUES.Tot	2.2	0.3	2.1	0.3	0.7	47	0.458
CPZ.EQ	6	2.9	5.5	3.5	0.7	49.9	0.493

For each of the 42 outcome variables, the table displays the sample mean and standard deviation of the variable over the Expe patients, then over the TR patients, then the results (test statistic, degrees of freedom, *p* value) of the Walsh two-sample two-sided *T* test between both. A *p* value smaller than 0.05 indicates that the difference of means is significant. CPZ100eq: dose equivalent to 100 mg/day of chlorpromazine calculated according to the minimum effective dose method [25]. The mean antipsychotic dose in the sample was equivalent to 6 mg/day chlorpromazine in the RR group and to 5.5 in the TR group.

Abbreviations: AcSo, self-assessment of social cognition; AIHQ, Ambiguous Intentions and Hostility Questionnaire (agress, aggression bias; anger, anger score; blame, blame score; host, hostility bias; resp, attribution of responsibility); BUES, Boston University Empowerment Scale; BEM-RI, BEM immediate recall; BEM-RD, BEM delayed recall; Coding, WAIS-IV Coding subscale; Education, Education level (years); ERF-CS, Social Cognition—Functional Outcomes Scale; ISMI, Internalized Stigma of Mental Illness scale (tot, total score; Alien, alienation subscale; stereo, stereotype endorsement; discri, discrimination experience; soc-with, social withdrawal; Rstig, stigma resistance); MASC, Movie for the Assessment of Social Cognition (Abs, absence of ToM; CM, correct mentalization score; Ho, hypomentalization; Hy, hypermentalization score); PANSS, Positive and Negative Syndrome Scale (positive, negative, and general psychopathology subscales); PerSo, social perception test; PSP, Personal and Social Performance Scale total score; QCAE, Questionnaire of Cognitive and Affective Empathy; SERS-SF, Self-Esteem Rating Scale-short form (positive and negative subscales); Simil NB, WAIS IV Similarities subtest; STORI, stage of recovery instrument (awar, awareness; growth, growth stage; mora, moratorium stage; prep, preparation stage; rebuil, rebuilding stage); TMT, Trail Making Test A and B; Bold values indicate the p-values significant at p<0.05.

### Outcome measures

#### Social functioning and social cognition

##### Social functioning (Personal and Social Performance Scale [PSP])

Social function was evaluated with the Personal and Social Performance Scale (PSP) [26]. PSP provides an overall rating score ranging from 1 to 100, higher scores representing better personal and social functioning. PSP showed acceptable internal consistency (Cronbach’s alpha [CA] = 0.76) [27], excellent inter-rater reliability (ICC = 0.97) [28], and a satisfactory ability to detect changes [26]. A seven-point improvement during clinical trials is considered to be clinically significant [26].

##### Theory of mind (Movie for the Assessment of Social Cognition (MASC)

The MASC test [29,30] is a 15mn movie depicting four people discussing on a Saturday evening. During the film the participant is regularly asked about the mental states of the characters with four possible options: (a) correct answer (ToM); (b) under-mentalizing answer; (c) lack of mental state attribution answer; and (d) over-mentalizing answer. A total score of correct answers and three error scores (less ToM, no ToM, and excessive ToM) are calculated. This scale has shown excellent internal consistency (CA = 0.865) [30].

##### Social perception and knowledge (PerSo)

The PerSo [31] measures competence in the perception of social situations depicted in four pictures taken from the material “ColorCards-Social Behavior.” Participants are firstly asked to describe all the elements in the picture allowing the calculation of global “fluency score.” Then participants describe the social situation, a total interpretation score (sum of the noncued and cued scores) being calculated. A score of social knowledge is calculated with a question concerning a social convention depicted on the card.

##### Attributional style (Ambiguous Intentions and Hostility Questionnaire [AIHQ])

The AIHQ [31,32] measures hostile social cognitive bias. Participant is asked to read each situation and to imagine it happening to him/her. Three scores are calculated depending on participant’s answers: hostility, attribution of responsibility, and aggression scores. This scale has shown excellent internal consistency (CA: 0.91–0.99) [32].

##### Empathy (Quotient of Cognitive and Affective Empathy [QCAE])

The QCAE [33,34] is a 31 item self-reported assessment comprising five subscales intended to assess cognitive and affective components of empathy. This scale has shown acceptable internal consistency (CA = 0.62–0.89) [34].

##### Self-assessment of social cognition impairments (ACSo)

Self-assessment of social cognition was realized with 12 items-ACSo questionnaire [31,35,36]. The ACSo showed acceptable internal consistency (CA = 0.822) [31].

##### Assessment of social cognition-related disability

Social cognition related disability was assessed with the 14-items Social Cognition-Functional Outcomes Scale (ERF-CS) [35]. The ERF-CS total score reflects the impact of social cognitive deficits on functional outcomes, a higher score being associated with increased severity [31]. This scale often used as a prepost measure for cognitive remediation, aims at determining one’s personal objectives and at facilitating the generalization of treatment benefits to daily life.

### Secondary outcomes

General information on education, marital status, economic status, illness onset and trajectory and comorbidities, was recorded. Illness severity was assessed using the Positive and Negative Syndrome Scale (PANSS) [37] with an adequate inter-rater reliability (ICC = 0.66). Self-stigma was assessed using the Internalized Stigma of Mental Illness scale (ISMI) [38], a 29-item self-report measure designed to assess people’s personal experience of stigma related to mental disorders. A higher score reflects a higher level of self-stigma. This scale has shown good internal consistency (CA = 0.80–0.92) [39] and test–retest reliability (0.92) [39]. Self-esteem was measured with the Self-Esteem Rating Scale-Short Form (SERS-SF; CA = 0.87–0.91) [40], empowerment with the Boston University Empowerment Scale (BUES; CA = 0.86) [41] and personal recovery using the self-reported Stage of Recovery Instrument (STORI) [42]. Neuropsychological baseline and follow-up cognitive assessments included Wechsler Adult Intelligence Scale-fourth edition (WAIS-IV) [43] Coding and Similarities subtests respectively for speed of processing and verbal abstraction, Trail Making Test A and B (TMT-A and B) [44], respectively for speed of processing and reactive mental flexibility, shopping test-revised for planning abilities [45], BEM-144 story subtest for auditory-verbal memory [46], and premorbid IQ with the French-National Adult Reading Test (f-NART) [47].

### Interventions

#### The experimental intervention

**Figure 1.**
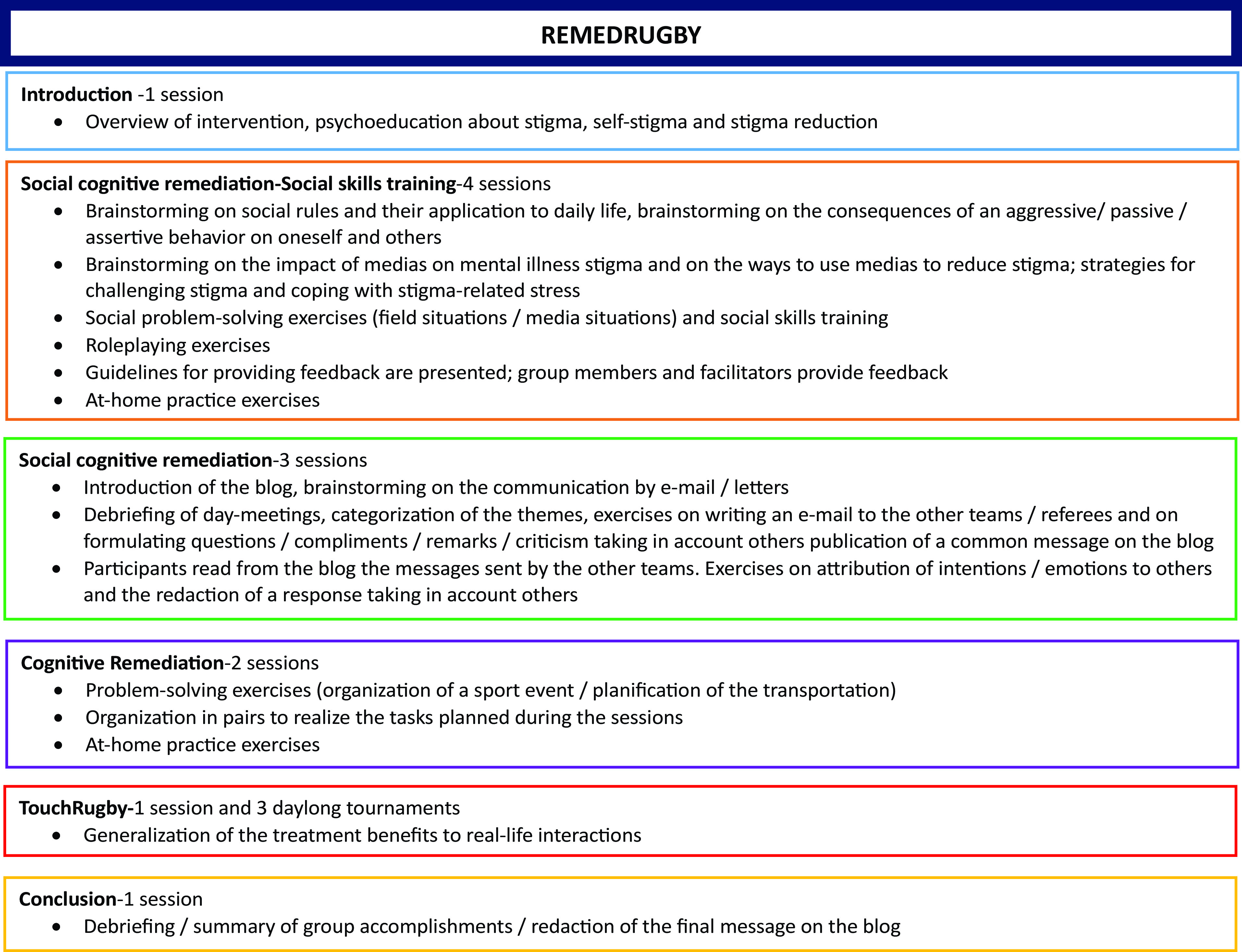
Overview of the RemedRugby topics.

RR is a manualized 15-session group-based intervention following the principles of cognitive remediation (“a behavioral training intervention targeting cognitive deficits using scientific principles of learning, with the ultimate goal of improving functional outcomes” [48]). Figure 1 provides an overview of the RR topics.

RR consists of 12 weekly 2-h sessions animated in the local rugby club by two facilitators and three daylong TR tournaments regrouping participants from all the centers. Sessions are conducted in a sequential fashion. In each session, participants are encouraged to generate information about the topic of the session and to remind the content of the previous session (freely and with cuing when necessary). The major part of each session is dedicated to the learning of a technique with its practical applications. Participants are invited to read the exercises from their manual, complete the prompts and to share them with other group members and to discuss their views related to them. They are invited to identify and monitor the strategies they use during the exercises and to consider how they could apply these strategies to their daily life. At the end of a session participants are encouraged to summarize what was learnt with its potential applications to their daily life. A part of each session (15 mn) and one specific session are dedicated to the practice of TR under facilitator’s supervision. At-home practice exercises are carried out between the sessions. RR has four major foci each of which targeting a specific domain: (a) SCR and SST applied to the situations that can be lived in the field (i.e., a referee not signaling a fault or a partner missing a pass), the following emotional possible reactions and their consequences on oneself and on his/her partner; (b) SCR via a blog common to all teams where participants are invited to formulate their remarks, questions, compliments, and criticism about the rules, the duration of matches, the organization of the previous or next tournament. After each tournament, group members are encouraged to list freely the themes they would like to discuss with the other teams, to categorize them and to complete the exercise from their manual (redaction of an e-mail addressing all the selected themes). Participants are invited to take other’s perspectives when writing their messages and to ask themselves about the intention of their interlocutor or the impact of their answer on others. Participants are encouraged to share their strategies and feelings with others and to formulate the team’s common answer on the blog; (c) Problem-solving skills via the organization of a daylong TR tournament regrouping all participants and facilitators (>80 persons). Three tournaments are organized during the program (one per experimental center). Participants from the inviting team are encouraged to consider the different steps of the organization of a sport event (material needed, number of guests and matches, starting and finishing hours, site preparation, and cleaning; guest reception) and their duration. The 23 steps of the organization are presented in disorder to the participants. After reordering them in the right chronological fashion, participants are invited to estimate the duration of each step and to proceed to a cumulative calculation aiming at determining the ending time of the tournament. Participants are then encouraged to list freely the material necessary to the organization and to order it in categories. They are invited to organize to themselves in pairs, each pair having specific tasks to realize during the day. The program is then published on the blog to inform the other teams of the details of the event. Group members from the other teams take note of the program and location of the tournament, estimate with a map the time needed for transportation and set an appointed leaving time; (d) SCR and SST applied to interaction with the medias. Group members are encouraged to reflect on stigma, self-stigma and the ways to reduce stigma. Participants are encouraged to share with other group members their personal stories about coping with stigma and to discuss the potential impact of a media intervention in changing people’s opinion about mental illness. They are invited to complete an exercise from their manual in which they imagine the questions a journalist could ask on the day of the tournament and write the answers they would like to make. Participants discuss their answers with the other group members and formulate the message they would like to pass to the press and the general public. They are encouraged to use problem-solving skills to plan the situation and to realize role-playing exercises. Role-playing trainings are made on the field (close to the real situation), one player playing the journalist, one the cameraman and the others being interviewed. Group members and facilitators provide feedback on the role-play. On the final tournament, participants who are willing to be interviewed can answer to local journalists with the objective to contribute to mental illness destigmatization.

#### The control condition: TR group

The control condition refers to the group attending to twelve 2-h sessions of TR under the supervision of specialized sport educators and to the three tournaments regrouping all patients. Sessions are similar to trainings than can be provided in a local sport club adapted to participant’s physical fitness.

### Statistical analysis

RR patients were evaluated at three visits V1, V2, and V3: V1 occurred before the treatment, V2 immediately after, V3 6 months later. The TR patients only had V1 and V2 after the same delay as RR patients. Forty-six variables were treated. The 42 score variables were evaluated at V1, V2, and V3 for RR patients, only V1 and V2 for the TR patients. A first question was to detect possible differences between the two populations of RR and TR patients over the 46 variables of interest at V1. This was done for the 45 continuous variables, using Welch two-sample *T* test. Results are shown on Table 1. For the discrete variable (gender) Fisher’s exact test was used, but no dependence was detected. The second question was to assess the effect of the treatment on the 57 RR patients at V2, and whether it had lasted until V3. For each of the 42 score variables, the values on the 57 RR patients at V1, V2, and V3 were compared two by two using a Welch’s two-sample *T* test, the side being chosen so that a small *p* value should correspond to improvement of the patient’s condition between successive visits. The *p* values are reported in Table 2. The third issue was to evaluate the effect of the treatment on the improvement of the different scores between V1 and V2. Improvement was defined as the difference between the values at V1 and V2, oriented so that a positive difference should correspond to an improvement of the patient’s condition. Each difference was adjusted by a linear regression over its confounding value at V0. The question was: is adjusted improvement higher among RR patients? Improvement in mean was tested by Welch’s two-sample *T* test for each of the 42 score variables. Results are shown on Table 3. A power analysis was performed using the R package “pwr” [50] based on Cohen [51]. For sample sizes of 37 and 50, an alpha level of 0.05, and an effect size (Cohen’s *d*) of 0.554 (see Table 3), the expected power of a two-sample *T* test is 0.68. It increases with the effect size, and is above 0.99 for an effect size greater than 1. The Shapiro–Wilk normality test was applied to all the tested samples. A False Discovery Rate correction was applied to each vector of *p* values, using the Benjamini–Yekutieli method, which is the most stringent, and also the most robust to possible dependencies. Referring to Table 3, all *p* values <0.001 were still <0.01 after FDR correction (results not reported here, available upon request). For the multivariate analysis, only adjusted improvements in the four PANSS scores, and the PSP score were considered. The values at V1 of the five variables of interest were considered as covariates. Five more covariates were added: age, gender, education level, and participation rate. Thus, improvement for each of the five variables of interest was tested against a set of 11 potentially explanatory factors, including the binary predictor: RR versus TR. All covariates were included in a linear regression model. A stepwise variable selection was performed using Bayes’ information criterion (BIC). *p* Values for the complete linear regression model are shown on Table 4. Data was analyzed using the R software, version 3.2.3 (R Core Team) [52]. The psych package version 1.5.8, was used Revelle [53]. The significance level was set at 0.05. Effect size (Cohen’s *d*) was calculated using package effsize [53]. Size effects inferior to 0.20 were considered as negligible, from 0.20 to 0.40 as small, from 0.40 to 0.60 as moderate, and superior to 0.60 as strong [53].

**Table 2.** Improvements in the RemedRugby group at post-treatment and follow-up

	Baseline post-treatment	Baseline follow-up	Post-treatment follow-up
PANSS.Tot	**<0.001**	**<0.001**	**0.018**
PANSS.Pos	**<0.001**	**<0.001**	0.101
PANSS.Neg	**<0.001**	**<0.001**	**0.038**
PANSS.GP	**<0.001**	**<0.001**	**0.023**
PSP	**<0.001**	**<0.001**	**0.006**
ERFCS.Tot	**<0.001**	**<0.001**	0.181
Simil.NB	**<0.001**	**<0.001**	**0.002**
BEMRI.NB	**0.008**	**0.003**	0.158
BEMRD.NB	**0.021**	**<0.001**	**0.036**
TMTA.Time	**0.006**	**0.001**	**0.02**
TMTB.Time	**0.043**	**0.008**	**0.04**
Coding.NB	**<0.001**	**<0.001**	**0.015**
PerSo.Tot	**0.047**	0.306	0.264
Shopping.time	0.178	0.142	0.257
MASC.MC	0.113	**<0.001**	**0.045**
MASC.Hy	0.07	0.119	0.32
MASC.Ho	0.689	0.05	**0.061**
MASC.Abs	0.06	**0.031**	0.5
f.NART	0.052	**0.005**	0.053
ACSo.Tot	0.176	0.227	0.261
SERS.posit	**0.039**	0.308	0.735
SERS.negat	0.294	**0.012**	**0.013**
QCAE.tot	0.492	0.318	0.206
ISMI.Tot	**<0.001**	**0.048**	0.981
ISMI.Alien	0.107	0.079	0.398
ISMI.Stereo	**<0.001**	**0.026**	0.999
ISMI.Discri	**0.005**	0.124	0.812
ISMI.Socwith	**0.007**	0.213	0.988
ISMI.Rstig	0.953	0.495	0.36
AIHQ.Host	0.148	**0.002**	0.07
AIHQ.Intent	0.428	0.339	0.89
AIHQ.Anger	0.152	0.288	0.744
AIHQ.Blame	0.228	0.135	0.828
AIHQ.Agres	0.098	**0.012**	0.718
AIHQ.AttRe	0.236	0.193	0.89
STORI.Mora	0.368	**0.089**	0.13
STORI.Awar	0.657	0.28	0.152
STORI.Prep	0.479	0.797	0.904
STORI.Rebuild	0.335	0.119	0.226
STORI.Growth	0.127	**0.019**	0.341
BUES.Tot	0.654	0.969	0.633
CPZ.EQ	**0.04**	**0.012**	0.082

For each of the 42 outcomes variable, the table displays the *p* values of three Walsh paired samples one-sided *T* tests: values at V1 against V2, then V2 against V3, then V1 against V3. In each case, the alternative has been chosen so that a small *p* value should correspond to improvement of the patient’s condition between consecutive visits. CPZ100eq: dose equivalent to 100 mg/day of chlorpromazine calculated according to the minimum effective dose method [49].

Abbreviations: AcSo, self-assessment of social cognition; AIHQ, Ambiguous Intentions and Hostility Questionnaire (agress, aggression bias; anger, anger score; blame, blame score; host, hostility bias; resp, attribution of responsibility); BEM-RD, BEM delayed recall; BEM-RI, BEM immediate recall; BUES, Boston University Empowerment Scale; Coding, WAIS-IV Coding subscale; Education, education level (years); ERF-CS, Social Cognition—Functional Outcomes Scale; ISMI, Internalized Stigma of Mental Illness scale (Alien, alienation subscale; discri, discrimination experience; Rstig, stigma resistance; stereo, stereotype endorsement; soc-with, social withdrawal; tot, total score); MASC, Movie for the Assessment of Social Cognition (Abs, absence of ToM; CM, correct mentalization score; Ho, hypomentalization; Hy, hypermentalization score); PANSS, Positive and Negative Syndrome Scale (positive, negative, and general psychopathology subscales); PerSo, social perception test; PSP, Personal and Social Performance Scale total score; QCAE, Questionnaire of Cognitive and Affective Empathy; SERS-SF, Self-Esteem Rating Scale-Short Form (positive and negative subscales); Simil NB, WAIS IV Similarities subtest; STORI, tage of recovery instrument (awar, awareness; growth, growth stage; mora, moratorium stage; prep, preparation stage; rebuil, rebuilding stage); TMT, Trail Making Test A and B; Bold values indicate the p-values significant at p<0.05.

**Table 3.** Univariate differences between V1 and V2

	RR (*n* = 57)		TR (*n* = 30)		Welsh two-sample *t* test			
Difference variable	*m*1	*sd*1	*m*0	*sd*0	*T*	*df*	PV	Cohen_d
PANSS.Tot	3.7	12.1	−7.2	9.8	4.4	65.1	**<0.001**	**0.961**
PANSS.Pos	0.8	3.8	−1.6	4.3	2.5	48.4	**0.009**	**0.594**
PANSS.Neg	1.2	4.4	−2.4	4.1	3.7	58.9	**<0.001**	**0.827**
PANSS.GP	1.8	5.5	−3.5	5	4.4	60	**<0.001**	**0.991**
PSP	4.4	9.3	−8.3	11.5	5.1	48.1	**<0.001**	**1.255**
ERFCS.Tot	2.2	18.5	−4.1	15.9	1.6	60.6	0.061	0.357
Simil.NB	0.6	2.8	−1	2.6	2.5	64.5	**0.008**	**0.554**
BEMRI.NB	−0.1	1.5	0.2	1.9	−0.9	49.8	0.823	−0.229
BEMRD.NB	−0.1	1.7	0.1	2	−0.4	54.8	0.647	−0.090
TMTA.Time	0.9	8	−1.5	16.4	0.8	37.3	0.226	0.206
TMTB.Time	−2.6	38.3	4.7	33	−0.9	63.1	0.808	−0.197
Coding.NB	−0.3	7.6	0.6	6	−0.6	72.2	0.73	−0.134
PerSo.Tot	0.5	4.2	−0.8	4.7	1.3	55.5	0.1	0.309
Shopping.time	−0.1	2.7	0.3	2.4	−0.7	68	0.755	−0.154
MASC.MC	−0.1	4.1	0.1	4.6	−0.2	53.3	0.587	−0.053
MASC.Hy	0.2	2.3	−0.3	3.2	0.8	44.3	0.214	0.205
MASC.Ho	−0.4	3.4	0.8	3.5	−1.5	56.2	0.934	−0.360
MASC.Abs	0.2	2.1	−0.3	2.8	0.8	46.3	0.216	0.199
f.NART	0.2	4.4	−0.3	4.9	0.5	55.3	0.315	0.115
ACSo.Tot	0.1	7.4	−0.2	6.6	0.2	66.9	0.435	0.037
SERS.posit	0.5	9.1	−0.7	9.2	0.6	61.6	0.286	0.133
SERS.negat	−0.2	6.1	0.4	7.6	−0.4	52.6	0.639	−0.088
QCAE.tot	−1.6	6.9	2.5	7.3	−2.5	59.1	0.992	−0.583
ISMI.Tot	0	0.3	0	0.3	1.1	64	0.141	0.250
ISMI.Alien	−0.1	0.5	0.1	0.5	−1.7	62.3	0.952	−0.395
ISMI.Stereo	0.1	0.4	−0.1	0.4	2.1	61.8	**0.019**	**0.495**
ISMI.Discri	0.1	0.5	−0.1	0.4	1.7	66.5	**0.047**	**0.389**
ISMI.Socwith	0	0.5	0	0.4	0.4	73.8	0.343	0.088
ISMI.Rstig	−0.1	0.3	0.1	0.5	−1.4	46.4	0.918	−0.358
AIHQ.Host	0	0.6	−0.1	0.8	0.8	46.2	0.224	0.195
AIHQ.Intent	0.1	0.9	−0.2	1.1	1.4	51.7	0.087	0.339
AIHQ.Anger	0	0.7	−0.1	1.2	0.3	41.3	0.364	0.092
AIHQ.Blame	0.1	1	−0.1	1.2	0.7	49.4	0.235	0.182
AIHQ.Agres	0.1	0.4	−0.2	0.5	2.5	47.4	**0.008**	0.627
AIHQ.AttRe	0.1	0.8	−0.1	1	0.9	46.2	0.176	0.239
STORI.Mora	−0.6	6.5	1	6.7	−1	58	0.841	0.240
STORI.Awar	0.4	5.4	−0.6	8	0.5	43.8	0.294	0.141
STORI.Prep	−0.5	7.5	0.8	8.1	−0.7	55.7	0.754	0.166
STORI.Rebuild	−0.5	6.5	0.8	6	−0.9	63	0.814	0.208
STORI.Growth	−0.5	8.8	0.7	5.8	−0.7	73.7	0.758	−0.151
BUES.Tot	0	0.3	−0.1	0.2	1.4	69.2	0.088	0.306
CPZ.EQ	0.1	0.8	−0.1	0.3	1.8	79.6	**0.039**	0.317

For each of the 42 outcome variables, improvement was defined as the difference between the value at V2 and V1, oriented so that a positive difference should correspond to an improvement of the patient’s condition. Each difference was adjusted by a linear regression over its confounding value at V1. The table displays the sample size mean and standard-deviation of the adjusted difference over the Expe patients, then over the TR patients, then the results (test statistic, degrees of freedom, *p* value) of the Walsh two-sample one-sided *T* test between both. A *p* value smaller than 0.05 indicates that the mean improvement over Expe patients is significantly higher. CPZ100eq: dose equivalent to 100 mg/day of chlorpromazine calculated according to the minimum effective dose method [49].

Abbreviations: AcSo, self-assessment of social cognition; AIHQ, Ambiguous Intentions and Hostility Questionnaire (agress, aggression bias; anger, anger score; blame, blame score; host, hostility bias; resp, attribution of responsibility); BEM-RD, BEM delayed recall; BEM-RI, BEM immediate recall; BUES, Boston University Empowerment Scale; Coding, WAIS-IV Coding subscale; Education, education level (years); ERF-CS, Social Cognition—Functional Outcomes Scale; ISMI, Internalized Stigma of Mental Illness scale (Alien, alienation subscale; discri, discrimination experience; Rstig, stigma resistance; stereo, stereotype endorsement; soc-with, social withdrawal; tot, total score); MASC, Movie for the Assessment of Social Cognition (Abs, absence of ToM; CM, correct mentalization score; Ho, hypomentalization; Hy, hypermentalization score); PANSS, Positive and Negative Syndrome Scale (positive, negative, and general psychopathology subscales); PerSo, social perception test; PSP, Personal and Social Performance Scale total score; QCAE, Questionnaire of Cognitive and Affective Empathy; SERS-SF, Self-Esteem Rating Scale-Short Form (positive and negative subscales); Simil NB, WAIS IV Similarities subtest; STORI, tage of recovery instrument (awar, awareness; growth, growth stage; mora, moratorium stage; prep, preparation stage; rebuil, rebuilding stage); TMT, Trail Making Test A and B; Bold values indicate the p-values significant at p<0.05.

**Table 4.** Multivariate analysis

	PANSS.Tot	PANSS.Pos	PANSS.Neg	PANSS.GP	PSP
expetau	**<0.001**	**0.002**	**<0.001**	**<0.001**	**<0.001**
Age1	0.235	0.688	0.088	0.167	0.132
Gender	0.442	0.739	0.231	0.326	0.104
Education	0.114	**0.032**	0.15	0.532	0.946
Participation	**0.015**	0.195	0.123	**0.006**	0.375
PANSS.Tot1	0.501	0.433	0.785	0.422	0.971
PANSS.Pos1	0.461	0.666	0.876	0.636	0.771
PANSS.Neg1	0.632	0.452	0.916	0.622	0.847
PANSS.GP1	0.385	0.424	0.557	0.318	0.901
PSP1	0.896	0.031	0.34	0.205	0.741
CPZ.EQ1	0.549	0.837	0.743	0.481	0.327
*P* value	0.084	0.262	**0.028**	0.072	0.318

The table displays results of the multivariate analysis for the adjusted differences (improvement) of the four PANSS scores, and the PSP score, successively considered as response variables. Eleven potentially explanatory factors are considered. They include the exposure to the treatment, four descriptive variables, the values at V1 of the five variables of interest, and the level of treatment CPZ.EQ. The first 11 rows give the *p* value of the contribution to the complete linear model of each explanatory variable. On the last row, the *p* value of the two-way a nova of the simple linear regression against the full model is displayed.

Abbreviations: PANSS, Positive and Negative Syndrome Scale; PSP, Personal and Social Performance Scale; Bold values indicate the p-values significant at p<0.05.

## Results

**Figure 2.**
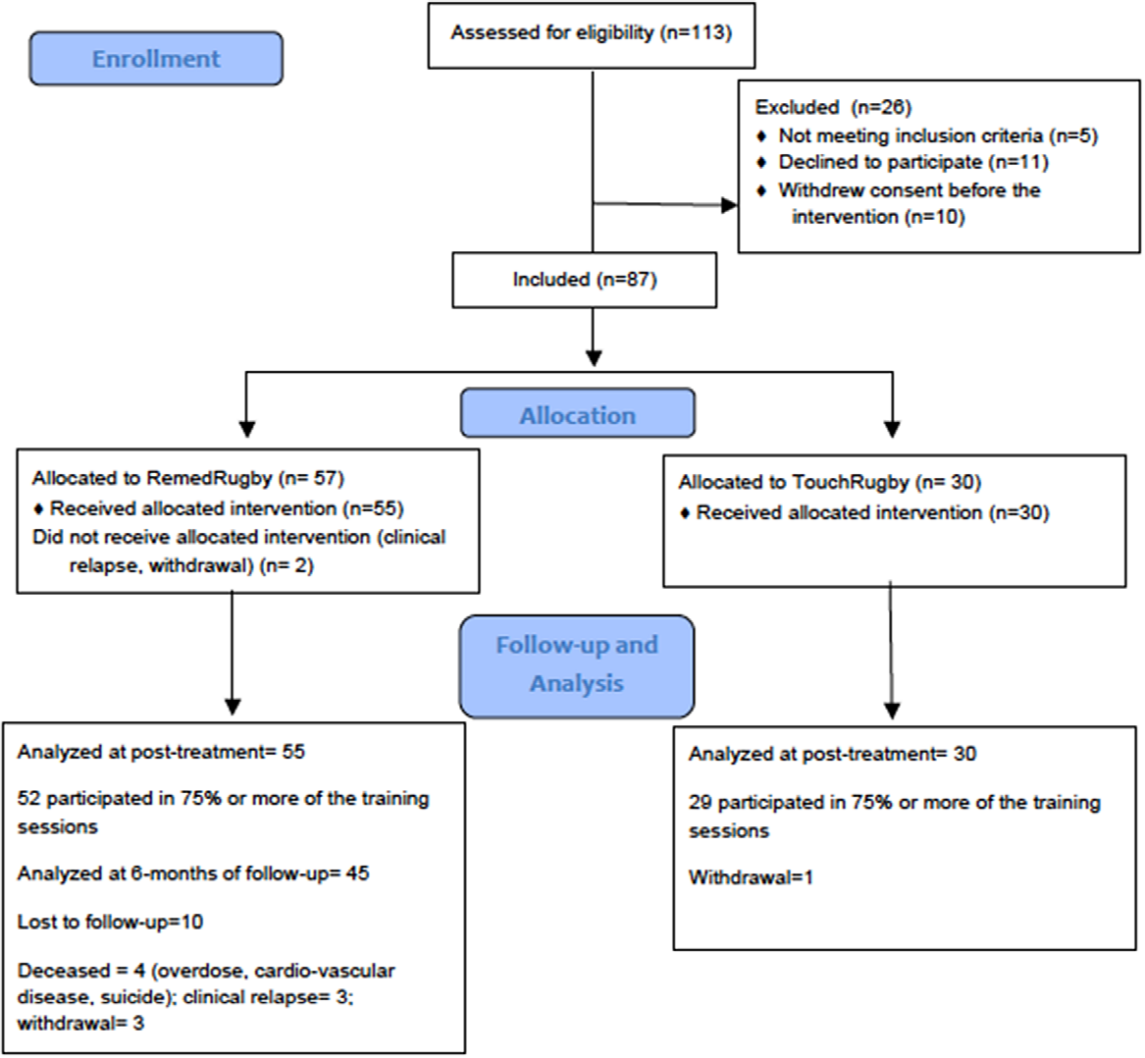
CONSORT flow diagram.

Figure 2 shows the CONSORT diagram. After excluding those who did not meet the inclusion criteria 57 participants were allocated to RR and 30 to the control condition. Baseline characteristics of the participants allocated to the two arms are presented in Table 1. The mean participation rate was 88% for those in the RR group and 93% for the control condition.

### Differences between the two groups before treatment

There were significant differences in MASC overmentalizing subscore (*p* = 0.024), PANSS total score (*p* = 0.022), negative symptoms (*p* = 0.004), and general psychopathology (*p* = 0.089) between the two groups before treatment. There were no significant baseline differences in pharmacological treatment, social function, neurocognition, self-esteem, self-stigma, empowerment, and personal recovery between the two groups.

### Changes in social functioning

After treatment RR participants showed larger improvements in social function (PSP; *p* < 0.001; *d* = 1.255) compared with TR patients.

### Changes in social and non-social cognition

RR participants improved more in aggression bias (AIHQ; *p* = 0.008; *d* = 0.627) and in social cognition-related disability (ERF-CS total, *p* = 0.061, *d* = 0.357; ERF-CS emotion processing, *p* = 0.011, *d* = 0.544; ERF-CS attribution style, *p* = 0.003; *d* = 0.624) than TR patients. RR participants showed larger improvements in verbal abstraction compared with TR patients (*p* = 0.008; *d* = 0554).

### Changes in symptom severity and other outcomes

RR participants showed larger improvements in symptom severity compared with TR group (PANSS total, *p* < 0.001; *d* = 0.961; PANSS positive, *p* = 0.009; *d* = 0.594; PANSS negative, *p* < 0.001; *d* = 0.827; PANSS GP; *p* < 0.001; *d* = 0.991). RR participants showed larger improvements in stereotype endorsement (*p* = 0.019, *d* = 0.495) and discrimination experiences (*p* = 0.047; *d* = 0.389) than TR patients. After treatment RR participants had lower antipsychotic doses than TR patients (*p* = 0.039; *d* = 0.317).

### Multivariate analysis

In the multivariate analysis, attending to RR was retained as the main explanatory factor for the improvement of social function (*p* < 0.001), positive (*p* = 0.002), negative (*p* < 0.001), and GP symptoms (*p* < 0.001) PANSS scores. Among the other 11 explanatory variables, few have a significant contribution. When comparing the model containing only the treatment with the full model with the 12 potential explanations, through a two-way ANOVA, the only response for which the full model does significantly better than the single one is PANSS negative (*p* = 0.028). For that variable the education level explanation improves on the treatment alone. For the other four responses, the treatment is indeed the best explanation by far, of the observed improvements.

## Discussion

The present study is the first trial to date evaluating the effectiveness of an integrated SCR program provided in a real-life environment. RR improved symptom severity and social function with large effect-sizes. RR improved moderately aggression social-cognitive bias, stereotype endorsement, discrimination experiences, social cognition-related disability, and verbal abstraction. The positive effects on these variables were specific to the RR group and were not observed in participants playing only TR. The effects on symptom severity, cognitive function and social functioning persisted after 6 months of follow-up. Participant’s age or gender and pharmacological or psychosocial treatments received during the study period had no influence on treatment’s outcomes.

According to Harvey et al.’s model [1], real-world functioning results from the interplay of cognitive performance, social cognition, functional capacity, negative symptoms, depression, environmental factors and physical fitness. Addressing simultaneously executive functioning, social competence, social cognition, negative symptoms, self-stigma and physical fitness may have improved treatment outcome on social functioning [1,2,11].

Negative symptoms including social amotivation and anhedonia are closely related with social functioning [1,2,11]. Social competence was associated with social motivation and was identified as a potential mediator of self-stigma effects on social functioning [2]. Improving social competence and social skills during treatment could make social interactions more enjoyable enhancing, therefore, the motivation to engage in social behavior [2,11,54]. Self-stigma is associated with impaired cognitive and social functioning, more severe positive and negative symptoms and increased social anxiety [55–62]. Reduced defeatist beliefs and self-stigma might contribute to the treatment effects on negative symptoms and social functioning [2,11,54]. Improvements in perceived social cognitive abilities might explain the treatment effects on self-stigma, psychiatric symptoms, and social functioning [63–67]. Improvements in self-stigma and hostile social-cognitive bias could explain the treatment effects on symptom severity, through reduced social anxiety and enhanced social motivation [2,61,62]. Cognitive remediation delivered in enriched environments [68,69] is effective on negative symptoms, possibly through improved executive functions [25]. Improvements in executive functions and in abstract reasoning, which has been related to functional capacity [70], might have contributed to the treatment effects on negative symptoms and social function. Contrary to our expectations, there was no improvement on objective measures of social perception and ToM. The improvement of social functioning and social-cognitive bias but not in social perception and ToM contrast with several other studies finding an opposite pattern of improvement after social-cognitive remediation (i.e., medium to large effects on ToM and social perception; small to medium effects on attribution bias; limited effects on social functioning [11]). The discrepancy between objective measures of social cognition and real-life functioning and the absence of consensus on an optimal set of social cognition outcome measures for clinical trials might explain these differences [11].

As above-mentioned aerobic PA showed effectiveness on patient’s outcomes in people with schizophrenia [17,18]. Effects on cognition were related to improvements in brain volumes and brain-derived neurotrophic factor (BDNF) [16,18]. Improvements in negative symptoms and social functioning after combining physical activity and cognitive remediation are in line with recent studies [16,71]. Increased physical fitness may have contributed to improved social function, but this variable was not measured during the study. Similarly, BDNF levels were not measured and there was no functional imaging. Future research should investigate whether the RR program could show effectiveness on these outcomes. Collective PA under the supervision of trained professionals (TR group) resulted in improved empathy and alienation subscale of self-stigma compared with the RR group. RR did not show effectiveness on personal recovery. To the best of our knowledge, this is the first study assessing these variables in the context of physical activity. Improvements in perceived social cognitive abilities could explain the absence of effects on alienation and personal recovery, through improved insight into illness [72]. The inclusion of more TR sessions in the RR program and a more explicit focus on recovery-related outcomes during the sessions might improve these outcomes. Unexpectedly physical activity alone resulted in aggravation in symptom severity and social function. One potential explanation to this discrepancy with the literature is that almost all studies provided individual activities [17,18]. Considering negative symptoms and social anxiety as barriers to PA participation [19–23] collective physical activities could be more challenging when provided alone and result in poorer outcomes at post-treatment.

In summary, an integrated exercise-enriched SCR program showed effectiveness in improving social functioning, aggression bias, symptom severity, verbal reasoning and self-stigma compared with the practice of a collective PA. Effects were persistent over time and even larger between post-treatment and follow-up. This indicates a potential generalization of treatment benefits to everyday functioning. The large sized effects on social function and psychopathology suggest that providing SCR in an enriched environment could be potentially beneficial in the treatment of people with schizophrenia by improving social function. Future research should investigate the potential effects of this intervention on neuroplasticity and physical fitness.

### Limitations

There are some limitations to be taken into account when interpreting the results of this study. The absence of randomization is an important limitation, possibly leading to potential selection bias and to baseline differences in psychopathology and social cognition. In addition, there was no follow-up after treatment in the TR group, making impossible to compare outcomes between the two groups at 6 months. One cannot therefore draw the conclusion that the significant improvements in the RR group between post-treatment and follow-up are caused by the participation to RR. Eventually, it is possible that some institutionalized or very disabled patients unfit to practice a physical activity according to their general practitioner’s examination, could not be able to participate in RR. Sites lacking of to an indoor or outdoor space where TR games can be practiced may also not be able to implement this intervention.

### Strengths

This is the first trial to date evaluating the effectiveness of an integrated SCR program provided in a real-life environment compared with an active control group. The present study exhibits clear strengths: the use of a large bundle of standardized evaluation scales, the absence of baseline differences in most of the variables considered, the possibility to rule out the effects of pharmacological and other psychosocial treatments on treatment’s outcomes and the inclusion of a large number of potential confounding factors in the multivariate analysis. TR is an intervention accessible to all types of participants regardless of their gender, age or physical fitness that can be practiced in nonspecific outdoor spaces or local sport clubs.

## Data Availability

The data that support the findings of this study are available on request from the corresponding author. The data are not publicly available due to privacy or ethical restrictions.
